# The effect of insulin degludec on risk of symptomatic nocturnal hypoglycaemia in adults with type 1 diabetes and high risk of nocturnal severe hypoglycaemia (the HypoDeg trial): study rationale and design

**DOI:** 10.1186/s12902-019-0408-x

**Published:** 2019-07-23

**Authors:** Rikke Mette Agesen, Amra Ciric Alibegovic, Henrik Ullits Andersen, Henning Beck-Nielsen, Peter Gustenhoff, Troels Krarup Hansen, Christoffer Hedetoft, Tonny Jensen, Claus Bogh Juhl, Susanne Søgaard Lerche, Kirsten Nørgaard, Hans-Henrik Parving, Lise Tarnow, Birger Thorsteinsson, Ulrik Pedersen-Bjergaard

**Affiliations:** 1 Department of Endocrinology and Nephrology, Nordsjællands Hospital, Hillerød, Dyrehavevej 29, DK-3400 Hillerød, Denmark; 2 Steno Diabetes Center Copenhagen, Niels Steensens Vej 2, DK-2820 Gentofte, Denmark; 3 Department of Endocrinology M, Odense University Hospital, Søndre Boulevard 29, DK-5000 Odense C, Denmark; 4 Faculty of Health Sciences, University of Southern Denmark, J.B. Winsløws Vej 19, 3, DK-5000 Odense C, Denmark; 5 Department of Endocrinology, Aalborg University Hospital, Mølleparkvej 4, DK-9000, Aalborg, Denmark; 6 Steno Diabetes Center Aarhus, Hedeager 3, DK-8200 Aarhus N, Denmark; 7 Health, University of Aarhus, Nordre Ringgade 1, DK-8000 Aarhus C, Denmark; 8 Department of Internal Medicine, Køge Sygehus, Lykkebækvej 1, DK-4600 Køge, Denmark; 9 Department of Medical Endocrinology, Copenhagen University Hospital (Rigshospitalet), Blegdamsvej 9, DK-2100 Copenhagen, Denmark; 10 Department of Medicine, Sydvestjysk Sygehus, Finsensgade 35, DK-6700 Esbjerg, Denmark; 11 Department of Diabetes and Hormonal Diseases, Lillebælt Hospital Kolding, Sygehusvej 24, DK-6000 Kolding, Denmark; 12 Department of Endocrinology, Hvidovre University Hospital, Kettegaards Alle 30, DK-2650 Hvidovre, Denmark; 13 Department of Clinical Research, Nordsjællands Hospital, Hillerød, Dyrehavevej 29, DK-3400 Hillerød, Denmark; 14 Steno Diabetes Center Sjaelland, Akacievej 7, DK-4300 Holbaek, Denmark; 15 Faculty of Health and Medical Sciences, University of Copenhagen, Blegdamsvej 3B, DK-2200 Copenhagen, Denmark

**Keywords:** Type 1 diabetes, Nocturnal hypoglycaemia, Severe hypoglycaemia, Insulin degludec, Insulin glargine, RCT

## Abstract

**Background:**

Hypoglycaemia, especially nocturnal, remains the main limiting factor of achieving good glycaemic control in type 1 diabetes.

The effect of first generation long-acting insulin analogues in reducing nocturnal hypoglycaemia is well documented in patient with type 1 diabetes. The effect of the newer long-acting insulin degludec on risk of nocturnal hypoglycaemia remains undocumented in patients with type 1 diabetes and recurrent severe nocturnal hypoglycaemia.

The HypoDeg trial is designed to investigate whether insulin degludec in comparison with insulin glargine U100 is superior in limiting the occurrence of nocturnal hypoglycaemia in patients with recurrent nocturnal severe hypoglycaemia. This paper reports the study design of the HypoDeg trial.

**Methods/design:**

A Danish investigator-initiated, prospective, randomised, open, blinded endpoint (PROBE), multicentre, two-year cross-over study investigating the effect of insulin degludec versus insulin glargine U100 on frequency of nocturnal hypoglycaemia in patients with type 1 diabetes and one or more episodes of nocturnal severe hypoglycaemia during the preceding two years as the major inclusion criteria. Patients are randomised (1:1) to basal therapy with insulin degludec or insulin glargine. Insulin aspart is used as bolus therapy in both treatment arms.

**Discussion:**

In contrast to most other insulin studies the HypoDeg trial includes only patients at high risk of hypoglycaemia. The HypoDeg trial will compare treatment with insulin degludec to insulin glargine U100 in terms of risk of nocturnal hypoglycaemic episodes in patients with type 1 diabetes with the greatest potential to benefit from near-physiological insulin replacement therapy. www.clinicaltrials.gov: NCT02192450.

## Background

Hypoglycaemia is the main side-effect of insulin therapy in type 1 diabetes and remains a major source of daily concern for patients and their relatives. Nocturnal hypoglycaemia, in particular, is a barrier against achievement of good glycaemic control as the fear of these episodes may impose inappropriate avoidance behaviour resulting in overnight hyperglycaemia in many patients [1]. Reduction of nocturnal hypoglycaemia in type 1 diabetes is therefore important to improve overall glycaemic control.

Improved basal insulin therapy has consistently resulted in reduced risk of nocturnal hypoglycaemia. Thus, the first-generation insulin analogues insulin glargine U100 and insulin detemir both reduce nocturnal hypoglycaemia by 26–53% compared to NPH insulin [2–4]. Insulin detemir reduces the risk of nocturnal (severe and non-severe) hypoglycaemia even in patients with type 1 diabetes and recurrent severe hypoglycaemia [5–8]. However, nocturnal hypoglycaemia remains a clinical challenge even with the use of these insulin analogues.

Insulin degludec is a newer long-acting basal insulin with a half-life of more than 24 h [9]. A recent study has demonstrated a significantly lower day-to-day variability than insulin glargine U100 – a coefficient of variation (CV) of 21% versus 83%, respectively [10]. Two phase 3 trials and a meta-analysis involving patients with type 1 diabetes have demonstrated lower rates of nocturnal hypoglycaemia with insulin degludec compared to insulin glargine U100 [11–13]. Like most other insulin trials, however, these trials only included participants at low risk of hypoglycaemia and accordingly the absolute risk reduction of nocturnal hypoglycaemia with insulin degludec is small. The results from the phase 3 programme have been confirmed in the SWITCH1 trial, which included patients with type 1 diabetes and increased risk of hypoglycaemia (e.g. people with impaired hypoglycaemia awareness, previous severe hypoglycaemia, or impaired renal function) [14]. The SWITCH1 trial did not specifically study people at particular risk of severe nocturnal hypoglycaemia, which may be those with the greatest potential to benefit from insulin degludec. The effect of insulin degludec in limiting nocturnal hypoglycaemia remains undocumented in this high-risk type 1 diabetes population.

The aim of this study is to compare the effect of insulin degludec with insulin glargine U100 on risk of nocturnal hypoglycaemia in patients with previous severe nocturnal hypoglycaemia.

## Aims

The *primary aim* is to test the hypothesis that treatment with insulin degludec as compared to insulin glargine U100 results in lower risk of symptomatic nocturnal hypoglycaemia in patients with type 1 diabetes and high risk of severe nocturnal hypoglycaemia.

The *secondary aims* are – with reference to the American Diabetes Association (ADA) criteria for classification of hypoglycaemia [15] – to investigate the effect of insulin degludec compared to insulin glargine U100 on:

- Any hypoglycaemic episode (symptomatic, asymptomatic/silent, severe) analysed according to severity and/or time of day
- Maintenance of baseline glycaemic control (HbA1c)
- Quality of life and depression/anxiety

## Methods/design

### Design, comparative treatment regimens and study duration

**Fig. 1:**
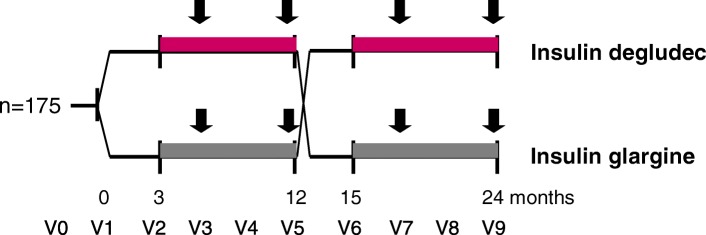
Study design.  Endpoint assessment periods.  Admission for overnight plasma glucose monitoring and/or 6 days of ambulatory 24 h continuous glucose monitoring

A Danish investigator-initiated, cross-over, multi-centre study conducted in a prospective, randomised, open, blinded endpoint (PROBE) design (Fig. 1). Patients are randomised to treatment with basal-bolus therapy with insulin degludec and insulin aspart (Tresiba®/NovoRapid®) or insulin glargine U100 and insulin aspart (Lantus®/NovoRapid®) in random order. Each treatment period lasts for 12 months. The first three months of each treatment period is a run-in or cross-over phase to be used for insulin dose adjustment and stabilisation of treatment regimens. Endpoints are assessed during the last nine months of each treatment arm (Fig. 1). Optional admissions for overnight plasma glucose measurements and six days of ambulatory blinded continuous glucose monitoring will be performed after 6 and 12 months in each treatment arm (Fig. 1).

Insulin doses will be adjusted according to the individual patient’s need at the discretion of the individual physician. Patients are instructed to perform and record 4-point self-monitored blood glucose (SMBG) profiles twice per week (before breakfast, before lunch, before dinner and at bedtime). A treat-to-target design is deliberately not used in order not to intervene with present glycaemic control and everyday clinical practice, which in itself may interfere with the risk of hypoglycaemia. We expect the individual patient to present the best obtainable glycaemic control with respect to risk of hypoglycaemia at inclusion, and we therefore strive for maintenance of baseline glycaemic control in both treatment periods. If, however, the glycaemic control is not acceptable we will strive for the best obtainable glycaemic control in both treatment periods, taking the risk of hypoglycaemia into account.

The HypoAna trial [5] showed significantly increased rates of severe hypoglycaemia during run-in and cross-over periods. Therefore, insulin doses are reduced at entrance into both treatment arms. A 20% reduction in both basal and bolus insulin dose is recommended when the patient changes from one insulin regimen to another, irrespective of the product. Rapid up titration by mandatory phone consultations weekly at week 1, 2 and 4 is followed by ad hoc titration at the investigators’ discretion.

The study is a once daily (QD) comparison between the insulins with long duration of action, i.e. degludec and glargine. Insulin degludec and insulin glargine U100 are administered subcutaneously in the thigh or in the abdominal wall with the evening meal. Correct injection technique is assured by a diabetes specialist nurse. The site of injection should be changed within the same anatomical region to avoid lipohypertrophy making insulin absorption more unpredictable.

The study was approved by The Regional Committee on Biomedical Research Ethics.

(#H-3-2014-101), the Danish Medicines Agency (#2014071615) and the Danish Data Protection Agency (I-suite no: 02945; #NOH-2014-018). The study is registered at www.eudract.ema.europe.eu (#2014–001942-24) and at www.clinicaltrials.gov (# NCT02192450). The Study is conducted in accordance with the Helsinki Declaration.

### Participants

#### Pre-screening/pre-selection

Pre-screening/pre-selection approaches are used at the participating outpatient clinics.

The primary approach is a questionnaire, which is mailed to the patients or completed by the patients in the clinic. Patients with one or more episodes of nocturnal severe hypoglycaemia in the preceeding two years are interviewed by telephone to validate severity (need for assistance from others) and causality of the events.

#### Screening, in- and exclusion criteria

Patients with one or more episodes of nocturnal severe hypoglycaemia during the last two years are screened (*n* = 154) according to in- and exclusion criteria.

The inclusion criteria are type 1 diabetes for more than five years, one or more episodes of nocturnal severe hypoglycaemia in the previous two years (defined as need for third party assistance to restore blood glucose level), age > 18 years, treatment with multiple dose insulin injection (more than 2) or insulin pump allowing for both human insulin and insulin analogues, a negative pregnancy test, willingness to a once-daily (QD) regimen concerning basal insulin, willingness to do SMBG and keep a diary.

Exclusion criteria are history of primary or secondary adrenal or growth hormone insufficiency, untreated hypothyroidism, history of unstable angina or major cardiovascular events, heart failure (New York Hart Association (NYHA) class IV), history of malignancy unless a disease-free period exceeding five years, history of alcohol or drug abuse, pregnancy or lactation, and women of childbearing potential who are not using chemical or mechanical contraception, HbA1c > 86 mmol/mol (10%), and shifting working hours. Criteria for discontinuation are withdrawal of consent, pregnancy or non-compliance with the study protocol as judged by the investigator. Discontinuing patients are not substituted, but data on discontinuing patients are included in data analyses on an intention-to-treat basis.

Participants were included in the HypoDeg Trial from January 2015 to February 2017. Last patient, last visit expected in February 2019.

### Randomisation

Patients are randomised 1:1 to insulin degludec or insulin glargine. Treatment allocation will be obtained by a web-based electronic case report file (CRF). The randomisation will be in blocks of four on site. Treatment allocation will not be blinded to patients or study personnel caring for patients since insulin analogues differ in therir pharmacokinetic properties.

### Visit procedures

Patients are seen in the outpatient clinics at Nordsjællands Hospital, Steno Diabetes Center Copenhagen, Hvidovre University Hospital, Køge Hospital, Odense University Hospital, Copenhagen University Hospital (Rigshospitalet), Sydvestjysk Sygehus, Frederica Hospital, Aalborg Hospital and Aarhus University Hospital for 10 visits.

Prior to any protocol-related procedures written informed consent is obtained. At visit 0 (V0) patients are screened for in- and exclusion criteria and eligible patients are scheduled for enrolment in the treatment phase of the study. Information about demography, lifestyle and medical history including diabetes treatment history, diabetic complications and concomitant medication, is gathered at the screening visit. Frequencies of episodes of nocturnal symptomatic hypoglycaemia during the last month and severe hypoglycaemia within the previous two years (overall and nocturnal) are reported at V0 [16]. The patient’s insulin injection technique is tested and corrected, if needed, and patterns of insulin adjustment are clarified. A physical examination including vital signs and body measurements completes the screening visit.

At randomization (V1) the patients attend the clinic after an overnight fast to enable fasting blood samples (glucose and C-peptide). Urinary albumin excretion rate is estimated in one overnight urine sample, and a biobank of serum, plasma, DNA and urine samples are established and stored at − 80 °C to permit subsequent analyses of newly discovered relevant markers without further discomfort to the patient.

Autonomic dysfunction is evaluated by the Vagus^tm^ device (Medicus Engineering, Aarhus, Denmark), which provides data on active tests of autonomic dysfunction following guidelines and recommendations of ADA and the European Association for the Study of Diabetes (EASD) [17, 18]. A 12-lead electrocardiogram is recorded and visually assessed. State of hypoglycaemia awareness is classified by three validated methods. [16, 19–21]. Four questionnaires about quality of life are filled in: The EuroQol-5 dimension quality of life index (EQ-5D-3 L), the Insulin Treatment Satisfaction Questionnaire (ITSQ), the Hypoglycaemic Confidence Scale (HCS) and the Hypoglycemia Fear Survey (HFS).

Two questionnaires about depression and anxiety are used: The Symptom Checklist (SCL-92) and Major Depression Inventory (MDI) [22–24].

The procedures performed at the randomisation visit (V1), except randomisation, are repeated at cross-over (V5) and at the end of the study (V9). At every visit (V1-V9) vital signs (blood pressure and pulse) and body measurements (weight) are recorded and HbA1c is measured.

## Endpoint registration

### Hypoglycaemia assessment

**Fig. 2:**
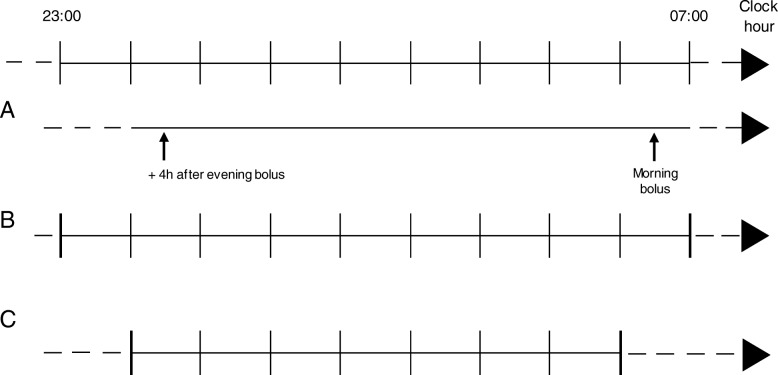
Definition of night. **a**: night is defined as time from 4 h after evening bolus of short acting insulin until time for actual morning bolus insulin administration. In the example shown the night is from 00:30 until 06:30, **b**: conventional definition of night from 23:00 to 06:59, **c**: conventional definition of night from 00:00 to 05:59

Hypoglycaemic events will be recorded during each treatment period – during night-time and during daytime. Night-time is defined from four hours after evening bolus of short-acting insulin until time for actual morning bolus insulin administration. Night-time will also be analysed conventionally from 23:00 to 06:59 and from 00:00 to 05:59 (Fig. 2). Daytime is defined conventionally from 07:00 to 22:59 and from 06:00 to 23:59. Data from the last nine months of each treatment arm will be used for endpoint analyses.

Hypoglycaemic endpoints are defined according to the latest ADA recommendations [15].

#### Documented mild symptomatic hypoglycaemia

Mild symptomatic hypoglycaemia is defined as an event during which typical symptoms of hypoglycaemia are accompanied by a measured plasma glucose concentration ≤ 3.9 mmol/l and treated without assistance from other persons. Patients are instructed and repeatedly encouraged to keep a diary on hypoglycaemia. Patients will report all events of symptomatic hypoglycaemia including date, time of episode, the accompanying measurement of plasma glucose, symptoms, awake or asleep, and time for last bolus insulin administration. In addition, all episodes occurring during night-time must be reported by telephone to a nurse within 24 h or first upcoming weekday after the event. All possible nocturnal symptomatic hypoglycaemic episodes will be adjudicated by an independent endpoint committee consisting of diabetes specialists blinded to the individual patient’s insulin regimen.

#### Asymptomatic hypoglycaemia

Asymptomatic hypoglycaemia is defined as an event not accompanied by typical symptoms of hypoglycaemia but with a measured plasma glucose concentration ≤ 3.9 mmol/l. Assessment of asymptomatic hypoglycaemia is accommodated in the 4-point SMBG profiles done twice a week and in the blinded CGM data obtained after 6 and 12 months in each treatment period.

Due to more recent hypoglycaemia guidelines [25] analyses of nocturnal symptomatic hypoglycaemia and any hypoglycaemic episode with a plasma glucose concentration ≤ 3.0 mmol/l are also planned.

#### Severe hypoglycaemia

Severe hypoglycaemia is a suspsected hypoglycaemic event requiring treatment assistance from another person. Carbohydrates, parenteral glucose or glucagon has to be actively administered to restore plasma glucose to normal. Plasma glucose measurements may or may not be available during such an event. The need of treatment assistance and adequate response to treatment is considered sufficient evidence that the event was induced by a low plasma glucose concentration. These episodes may be associated with sufficient neuroglycopenia to induce seizure or coma [15]. The patient must report all events of severe hypoglycaemia within 24 h by telephone to a nurse. Like in daily clinical practice investigators must encourage patients to report all episodes of severe hypoglycaemia at the quarterly visits at the outpatient clinics, hereby minimizing the risk of missing events.

A structured interview questionnaire will be applied to all possible severe hypoglycaemic episodes in order to validate episodes according to Whipple’s triad [26]. All episodes will be adjudicated by an independent endpoint committee consisting of diabetes specialists blinded to the individual patient insulin regimen.

### Additional hypoglycaemia assessment

#### In-hospital overnight glucose profiles

Patients are invited (optional) to stay overnight at Nordsjællands Hospital Hillerød four times during the study (after 6 and 12 months in each treatment arm). At the evening of the overnight stay the participants take part of a standard meal. Afterwards the participants are accommodated, and a venous line is inserted in an antecubital vein. During the night, blinded samples for subsequent plasma glucose measurements are drawn every hour from the catheter, while the patient is asleep [27].

#### Continuous glucose monitoring (CGM)

Optionally interstitial glucose concentrations are assessed twice for six days in each treatment arm using the CGM device iPro®2 digital recorder, MMT-7741 (iPro2) with the Enlite® sensor (MMT-7008 (Glucose sensor), Medtronic Minimed, Northridge, USA). The sensor is mounted by a trained study nurse and the patient is instructed how to calibrate the device, using capillary glucose measurements. The device is mounted in connection with visit 3, 5, 7 and 9 (after 6 and 12 months in each treatment arm).

After six days the glucose recorder is collected, and data are uploaded into CareLink iPro Therapy Management Software for Diabetes (CareLink iPro, MMT-7340) to generate reports and store data. These data remain blinded to the investigators until the end of the study and are not used to control glycaemic levels during the study period.

### HbA1c assessment

At every visit to the outpatient clinic (excluding V0) blood samples are drawn to measure HbA1c levels (analysed centrally at Nordsjællands Hospital).

## Statistics

### Power considerations

Rates and distribution of hypoglycaemic events are much skewed and unpredictable [6, 28]. The occurrence of hypoglycaemia is influenced by many factors, and by engaging in a study rates of hypoglycaemia usually are affected considerably.

Formal power calculations are therefore, not found feasible.

According to the power calculation and results from the HypoAna trial with a significant relative difference between treatment regimens of 30–40% concerning both primary and secondary nocturnal endpoints [5, 6, 8], we decided to include and randomise approximately 175 type 1 diabetes patients prone to severe hypoglycaemia (i.e. one or more episodes of nocturnal severe hypoglycaemia during the preceding two years).

### Planned analyses

Intention-to-treat and per-protocol statistical analyses will be conducted by the investigators, in close collaboration with a statistician experienced in analysis of hypoglycaemia data. Before the randomisation code will be added to the cleaned dataset, a thorough plan for statistical analyses will be elaborated and accepted by the statistician and investigators. The cross–over design makes it possible to accommodate the individual patient’s event parameters and continuous parameters. The per-protocol analyses will include only patients who have completed the first treatment period and at least 6 months of the second treatment period (cross-over plus three months maintenance).

Endpoints will be compared between treatment groups as an analysis of differences in rates during the last nine months of each treatment arm. In the simplest model this difference is assumed to be normally distributed and tested as such under the assumption that the HbA_1c_ levels are comparable during the two treatment arms. If necessary a more specific analysis in a negative binomial distribution model and/or a Poisson model will be used to describe the number of events through a suitable link function as a linear function of individual patient levels, treatment effects, treatment period, and mean HbA_1c_ level during endpoint assessment. Individual differences in the observation period will be adjusted for in the analyses.

For continuous parameters a co-variance analysis will be conducted.

The level of statistical significance is chosen as *p* < 0.05 (two-sided).

### Additional statistics

The obtained glycaemic control during the two treatment regimens will be evaluated through comparisons of HbA_1c_ levels after 12 months’ treatment including factors corresponding to treatment, treatment period and the individual patient level. Furthermore, SMBG profiles will be evaluated in addition to quantification of glucose variability during night-time and 6 days of CGM.

## Laboratory analyses

HbA1c is measured at every visit (V1-V9). At V1 (randomisation), V5 (cross-over) and V9 (end of study) haemoglobin, haematocrit, plasma creatinine, plasma sodium, plasma potassium, total cholesterol, high and low-density lipoprotein cholesterols, triglycerides, and thyroid-stimulating hormone (TSH), T4 and T3 are measured.

Fasting C-peptide is only measured at randomisation (V1) and concentrations below 20 pmol/l (= 0.02 nmol/L) are considered C-peptide negative.

Urinary albumin excretion rate is determined in one overnight urine sample at V1 and repeated after one year at V5 and after 2 years at V9. This also applies to the biobank samples (serum, plasma, DNA and urine) which are gathered at V1, V5 and V9.

HbA1c is measured centrally at Nordsjællands Hospital Hillerød using High Performance Liquid Chromatography method on a Tosoh Automated Glycohemoglobin Analyzer.

All other analyses are performed locally by routine methods.

## Discussion

In contrast to almost all other studies in this field the HypoDeg trial includes only patients who are hypoglycaemia-prone. The HypoDeg trial will elucidate whether once-daily insulin degludec in comparison with once-daily insulin glargine U100 during basal-bolus therapy is advantageous in limiting the number of hypoglycaemic episodes in these patients who may have the greatest benefit from optimized basal insulin therapy.

This insulin trial is one of a few to systematically evaluate the risk of hypoglycaemia by simultaneous adjudication of events, reporting in diaries and observation by CGM [5–7].

The results may have a direct evidence-based impact on the patients and their health care professionals in choice of basal therapy.

## Data Availability

The datasets used and/or analysed during the current study are available from the corresponding author on reasonable request.

## Abbreviations

ADA: American Diabetes Association
CGM: Continuous glucose monitoring
CRF: Case Report File
CV: Coefficient of Variation
EASD: European Association for the Study of Diabetes
EQ-5D-3 L: EuroQol-5 dimension quality of life index
HbA1c: Haemoglobin A1c
HCS: Hypoglycaemia Confidence Scale
HFS: Hypoglycemia Fear Survey
ITSQ: Insulin Treatment Satisfaction Questionnaire
MDI: Major Depression Inventory
NPH: Neutral protamine Hagedorn
NYHA: New York Heart Association
PROBE: Prospective, randomised, open, blinded endpoint
QD: quaque die (one daily)
SCL-92: Symptom Checklist
SMBG: Self-monitored blood glucose
TSH: Thyroid-stimulating hormone

## References

[1] Brod M, Wolden M, Christensen T, Bushnell DM (2013). A nine country study of the burden of non-severe nocturnal hypoglycaemic events on diabetes management and daily function. Diabetes Obes Metab.

[2] Monami M, Marchionni N, Mannucci E (2009). Long-acting insulin analogues vs. NPH human insulin in type 1 diabetes. A meta-analysis. Diabetes Obes Metab.

[3] Singh SR, Ahmad F, Lal A, Yu C, Bai Z, Bpharm HB (2009). Efficacy and safety of insulin analogues for the management of diabetes mellitus: a meta-analysis. CMAJ.

[4] Gough SCL (2007). A review of human and analogue insulin trials. Diabetes Res Clin Pract.

[5] Pedersen-Bjergaard U, Kristensen PL, Beck-Nielsen H, Nørgaard K, Perrild H, Christiansen JS (2014). Effect of insulin analogues on risk of severe hypoglycaemia in patients with type 1 diabetes prone to recurrent severe hypoglycaemia (HypoAna trial): a prospective, randomised, open-label, blinded-endpoint crossover trial. Lancet Diabetes Endocrinol.

[6] Agesen RM, Kristensen PL, Beck-Nielsen H, Nørgaard K, Perrild H, Christiansen JS (2016). Effect of insulin analogues on frequency of non-severe hypoglycaemia in patients with type 1 diabetes prone to severe hypoglycaemia: the HypoAna trial. Diabetes Metab.

[7] Agesen RM, Kristensen PL, Beck-Nielsen H, Nørgaard K, Perrild H, Jensen T (2018). Effect of insulin analogs on frequency of non–severe hypoglycemia in patients with type 1 diabetes prone to severe hypoglycemia: much higher rates detected by continuous glucose monitoring than by self-monitoring of blood glucose—the HypoAna trial. Diabetes Technol Ther.

[8] Kristensen PL, Tarnow L, Bay C, Nørgaard K, Jensen T, Parving H-H (2017). Comparing effects of insulin analogues and human insulin on nocturnal glycaemia in hypoglycaemia-prone people with type 1 diabetes. Diabet Med.

[9] Goldman-Levine JD, Patel DK, Schnee DM (2013). Insulin degludec: a novel basal insulin analogue. Ann Pharmacother.

[10] Heise T, Kaplan K, Haahr HL (2018). Day-to-day and within-day variability in glucose-lowering effect between insulin Degludec and insulin glargine (100 U/mL and 300 U/mL): a comparison across studies. J Diabetes Sci Technol.

[11] Heller S, Buse J, Fisher M, Garg S, Marre M, Merker L (2012). Insulin degludec, an ultra-longacting basal insulin, versus insulin glargine in basal-bolus treatment with mealtime insulin aspart in type 1 diabetes (BEGIN basal-bolus type 1): a phase 3, randomised, open-label, treat-to-target non-inferiority trial. Lancet.

[12] Bode BW, Buse JB, Fisher M, Garg SK, Marre M, Merker L (2013). Insulin degludec improves glycaemic control with lower nocturnal hypoglycaemia risk than insulin glargine in basal-bolus treatment with mealtime insulin aspart in type 1 diabetes (BEGIN ® basal-bolus type 1): 2-year results of a randomized clinical trial. Diabet Med.

[13] Ratner RE, Gough SCL, Mathieu C, Del Prato S, Bode B, Mersebach H (2013). Hypoglycaemia risk with insulin degludec compared with insulin glargine in type 2 and type 1 diabetes: a pre-planned meta-analysis of phase 3 trials. Diabetes Obes Metab.

[14] Lane W, Bailey TS, Gerety G, Gumprecht J, Philis-Tsimikas A, Hansen CT (2017). Effect of insulin Degludec vs insulin glargine U100 on hypoglycemia in patients with type 1 diabetes. JAMA.

[15] Seaquist ER, Anderson J, Childs B, Cryer P, Dagogo-Jack S, Fish L (2013). Hypoglycemia and diabetes: a report of a workgroup of the American Diabetes Association and the Endocrine Society. Diabetes Care.

[16] Pedersen-Bjergaard U, Pramming S, Thorsteinsson B (2003). Recall of severe hypoglycaemia and self-estimated state of awareness in type 1 diabetes. Diabetes Metab Res Rev.

[17] Rydén L, Standl E, Bartnik M, Van Den Berghe G, Betteridge J, De Boer MJ (2007). Guidelines on diabetes, pre-diabetes, and cardiovascular diseases: full text. Diabetes, Stoffwechsel Und Herz.

[18] Boulton AJM, Al VAIAJE (2005). Diabetic neuropathies: a statement by the American Diabetes Association. Diabetes Care.

[19] Clarke WL, Cox DJ, Gonder-Frederick LA, Julian D, Schlundt D, Polonsky W (1995). Reduced awareness of hypoglycemia in adults with IDDM. A prospective study of hypoglycemic frequency and associated symptoms. Diabetes Care.

[20] Gold AE, MacLeod KM, Frier BM (1994). Frequency of severe hypoglycemia in patients with type I diabetes with impaired awareness of hypoglycemia. Diabetes Care.

[21] Høi-Hansen T, Pedersen-Bjergaard U, Thorsteinsson B (2009). Classification of hypoglycemia awareness in people with type 1 diabetes in clinical practice. J Diabetes Complicat.

[22] Euroqol website n.d. https://euroqol.org/eq-5d-instruments/eq-5d-3l-about/ (accessed June 6, 2019).

[23] Anderson RT, Skovlund SE, Marrero D, Levine DW, Meadows K, Brod M, et al. Development and validation of the insulin treatment satisfaction questionnaire 2004:565–78. 10.1016/s0149-2918(04)90059-8 15189754

[24] Polonsky William H., Fisher Lawrence, Hessler Danielle, Edelman Steven V. (2017). Investigating Hypoglycemic Confidence in Type 1 and Type 2 Diabetes. Diabetes Technology & Therapeutics.

[25] International Hypoglycaemia Study Group. Glucose concentrations of less than 3.0 mmol/l (54 mg/dl) should be reported in clinical trials: a joint position statement of the American Diabetes Association and the Europian Association for the Study of diabetes. Diabetologia. 2017;60:3–6. 10.1007/s00125-016-4146-6. 10.1007/s00125-016-4146-6 PMC6518070 27872948

[26] Cryer PE. Defining and reporting hypoglycemia in diabetes: a report from the American diabetes association workgroup on hypoglycemia. Diabetes Care. 2005;28:1245–9. 10.2337/diacare.28.5.1245. 10.2337/diacare.28.5.1245 15855602

[27] Pramming S, Thorsteinsson B, Bendtson I, ROnn B, Binder C (1985). Nocturnal hypoglycaemia in patients receiving conventional treatment with insulin. Br Med J (Clin Res Ed).

[28] Kristensen PL, Hansen LS, Jespersen MJ, Pedersen-Bjergaard U, Beck-Nielsen H, Christiansen JS (2012). Insulin analogues and severe hypoglycaemia in type 1 diabetes. Diabetes Res Clin Pract.

